# Dietary Knowledge among Adults with Type 2 Diabetes—Kingdom of Saudi Arabia

**DOI:** 10.3390/ijerph17030858

**Published:** 2020-01-30

**Authors:** Waqas Sami, Khalid M Alabdulwahhab, Mohd Rashid Ab Hamid, Tariq A. Alasbali, Fahd Al Alwadani, Mohammad Shakil Ahmad

**Affiliations:** 1 Department of Community Medicine & Public Health, College of Medicine, Majmaah University. Al-Majmaah University, Al-Majmaah 11952, Saudi Arabia; m.shakil@mu.edu.sa; 2 Department of Ophthalmology, College of Medicine, Majmaah University. Al-Majmaah University, Al-Majmaah 11952, Saudi Arabia; k.alabdulwahhab@mu.edu.sa; 3 Centre for Mathematical Sciences, Universiti Malaysia Pahang, Lebuhraya Tun Razak, Gambang, Kuantan, Pahang 26300, Malaysia; rashid@ump.edu.my; 4 Department of Ophthalmology, College of Medicine, Al-Imam Mohammad Ibn Saud Islamic University, Riyadh 7544, Saudi Arabia; taalasbali@imamu.edu.sa; 5 Department of Ophthalmology, College of Medicine, King Faisal University, Hofuf 31982, Saudi Arabia; dr_wadani@yahoo.com

**Keywords:** dietary knowledge, type 2 diabetes, nutrition intake, Kingdom of Saudi Arabia

## Abstract

Dietary management is considered as a major step in assessing a patient’s knowledge related to nutritional aspects, treatment, and complications of diabetes. Diabetes patients frequently face difficulty in identifying the recommended diet, including its quality and quantity. In the Kingdom of Saudi Arabia (KSA), sedentary lifestyle, along with food choices and portion sizes, have increased considerably and this has resulted in the soaring risk of diabetes. In addition, there is paucity of literature focusing on the Dietary Knowledge (DK) of type 2 diabetics in KSA. The study aimed to assess and evaluate the DK of type 2 diabetics. An analytical cross-sectional study was conducted among 350 type 2 diabetics using a valid and reliable self-prepared questionnaire comprising of 21 questions. Results showed that type 2 diabetics had an overall poor DK (28.57%). Sub-group analysis further revealed that diabetes patients had poor knowledge related to the consumption of carbohydrates and food choices, whereas they had good knowledge related to lipids and fats, proteins and food types. The role of diet in controlling of diabetes is considered imperative, but still, diabetes patients are unaware how they should approach this issue. The patient empowerment approach can be used to counsel patients with a poor DK. Primary care physicians and dietitians should work together and carry out individualized, tailored and patient-centered dietary education sessions.

## 1. Introduction

Dietary Knowledge (DK) is the knowledge that deals with the process and concepts related to health and diet, disease and diet, the nutritional value of the foods the foods that explain the nutrients within them and the recommendations that should be followed [1,2]. The Centers for Disease Control and Prevention have identified self-dietary management as a major step in assessing a patient’s knowledge related to the nutritional aspects, treatment, and complications of diabetes [3]. Diabetes patients frequently face difficulty in identifying the recommended diet, including its quality and quantity. Food selection and dietary pattern are influenced by a patient’s knowledge related to a recommended diet [4]. The role standing of diet in controlling of diabetes is considered imperative; still, diabetes patients are unaware of how they should approach this issue to ensure good glycemic control [5]. DK has been identified as a significant factor that influences dietary behaviors [6].

A study conducted on type 2 diabetics concluded that areas zones related to DK, glycemic control and use of insulin had evident knowledge gaps. Addressing these gaps can eventually help in preventing diabetes complications [7]. The results of another study [8] showed that males had a significantly higher consumption of rice, read meat and fried food compared to females. Though it has been stated that having strong knowledge about the recommended diabetic diet significantly influences a patients’ food selection and dietary behaviors [9].

Recently, in the Kingdom of Saudi Arabia (KSA), sedentary lifestyle, along with food choices and portion sizes, have increased considerably that have resulted in the soaring risk of obesity. The increase in obesity has also been attributed to the easy availability of fast food [10]. Results of a study conducted in KSA [11] reported that more than 50% of diabetes patients refused to modify their dietary pattern, reduce their weight and be physically active, i.e., perform exercise. There is paucity of literature focusing on DK of type 2 diabetics in KSA. To the best of our knowledge this is the first study of its kind. Therefore, this study planned to assess and evaluate the DK of type 2 diabetics.

## 2. Materials and Methods

This was an analytical cross-sectional study by design, which was conducted among type 2 diabetes patients, February–April, 2017. The data from 350 patients were collected using a systematic random sampling technique via a direct investigation method. One sample proportion formula was used to calculate the sample size, keeping in view the prevalence of diabetes mellitus (23.7%) in KSA [12]. However, it was increased to 350, keeping in mind a number of factors (generalizability of results, missing data, withdrawing of patients and existence of outliers, if any). Inclusion criteria were—known to have type 2 diabetes; aged between 35-55 years; either gender, and patients having no co-morbidities. Written informed consent was taken from patients prior to data collection and anonymity was maintained. This research was approved by the ethical review committee of Majmaah University, KSA vides reference no: MURECApril.02/COM-2016.

The self-prepared Dietary Knowledge questionnaire (DKQ) used in this research comprised of 21 multiple choice questions (MCQs) (Supplementary Materials) that was prepared after intensively reviewing the literature. A psychometric analysis of the English version DKQ used in this research showed good internal consistency reliability (Cronbach Alpha = 0.783). Further details can be retrieved from [13]. The questions were designed to assess a patient’s DK about carbohydrates, lipids, proteins, food type and food choices. Each MCQ had only one correct answer. The responses were coded as 1 = correct and 0 = wrong, and I do not know. The score ranges from 0–21; a higher level of DK was indicated by a higher score. Furthermore, the quantitative score variable was categorized in percentage. A score <50% was considered as having poor DK, whereas, a score between 50%–75% was considered as having good DK, and a score >75% was considered as having adequate DK [14,15,16]. The DKQ was also translated into the Arabic language by an expert using the back-translation technique. This was done, in case patients had difficulty in understanding the English version of the questionnaire.

IBM SPSS version 25 (IBM Corp., Armonk, NY, USA) was used to analyze the data. A one-sample Kolmogorov Smirnov (K–S) test was applied to check the normality of quantitative variables. Detection of outliers was done through the Z-Scores univariate method. Frequencies and percentages were reported for qualitative variables, whereas, non-normally distributed variables were expressed as medians and quartiles (25th–75th). A one-sample Wilcoxon signed rank test was applied to evaluate the DK of type 2 diabetics. A Mann–Whitney U test was applied to compare DK scores between males and females. Spearman rho correlation was applied to observe correlations between BMI, duration of diabetes, and DK score. A Kruskal–Wallis H test was also applied to compare the DK scores among treating doctors. A p-value of less than 0.05 was considered statistically significant.

## 3. Results

The average age of the patients was 45 (40–51) years. Most of the patients were males (57.7%), compared to females (42.3%). More than 90% of the patients were married. Results presented in Table 1 show that more than (50%) of the patients’ duration of diabetes was between 5–10 years; around one third of the patients (35.1%) had diabetes less than 5 years, and only 9.4% had diabetes 11–15 years or longer. The mode of diagnosis for most patients was during a routine check-up (69.1%), at screening (12.6%), symptomatic (10.9%) and in an emergency (7.4%). The majority of patients were treated by general practitioners (74.9%), almost one fifth by physicians (21.1%) and a few (4%) by endocrine specialists. The family history of diabetes was positive in 40.6% of the patients. The majority of patients were non-smokers (53.4%), followed by smokers (40.3%), and never-smokers were only 6.3%. Almost 50% of the patients were overweight, followed by obese (32.9%). Patients having a normal weight were 16%, and only 3.4% of the patients were underweight.

Outlier detection results showed that all z-score values in DK score variable were less than the absolute value of 4. The minimum z-score value was -2.189 and maximum was 3.011. Therefore, no outlier problem was detected in the DK score variable. The average DK score out of 21 items was 6 (5–8). Minimum DK score was 1 and maximum was 15. In terms of percentage, type 2 diabetics had poor DK (28.57%). The result of a one-sample Wilcoxon signed rank test confirmed that type 2 diabetics had overall poor DK (*p* < 0.001). This was achieved by comparing the DK percentage score (28.57%) with the threshold value of 50%. The percentages of correct answers for each item are presented in Table 2. No significant difference was observed when DK score was compared between male and female diabetics using Mann–Whitney U test (*p* = 0.497). However, a significant inverse correlation was observed between DK score and BMI, showing that, as BMI increases, the DK score decreases, and vice versa (rho = 0.106, *p* = 0.048). No significant difference was observed when DK score was compared among treating doctors (χ^2^(2) = 1.749, *p* = 0.417) and with duration of diabetes (rho = 0.081, *p* = 0.131).

Items measuring DK were further categorized into sub-groups to assess the degree of knowledge of patients in specific areas. The sub-groups are carbohydrates (six items), lipids and fats (four items), proteins (two items), food type (six items) and food choices (three items). The median knowledge score of patients in carbohydrates group was 2 (0–3), which showed that type 2 diabetics had poor knowledge (33.3%) regarding carbohydrates. The median knowledge score of lipids and fats group was 2 (1–2); in percentages, it showed that type 2 diabetics had good knowledge (50%) regarding lipids and fats. The median knowledge score of protein group was 1 (0–3); this showed that type 2 diabetics had good knowledge (50%) regarding proteins. The median knowledge score of food type group was 3 (1–4). In terms of percentage, type 2 diabetics had good knowledge (50%) regarding food type. The median knowledge score of food choice group was 1 (0–2), which showed that type 2 diabetics had poor knowledge (33.33%) regarding food choices. Percentages of correct answers for each sub-group are presented in Table 3.

## 4. Discussion

In our research, type 2 diabetics had poor DK (28.57%). We also compared the DK scores between male and female diabetics, and the results showed a non-significant difference (*p* = 0.497). A significant inverse correlation was observed between DK score and BMI (*p* = 0.048); however, DK score, when correlated with duration of diabetes, showed no significance (*p* = 0.131). In addition, no significant difference was observed when DK score was compared among treating doctors (*p* = 0.417). There is a scarcity of literature comparing the DK scores of type 2 diabetics by gender, duration of diabetes, and treating doctors, so these results could not be compared. A study conducted in Iran [17] reported that type 2 diabetics had average knowledge regarding dietary management. Comparing our study results with those of the Iranian study, our patients also had poor DK. A study done by El-Qudah [15] revealed an inadequate DK in diabetes patients. Furthermore, the author recommended to set up health education programs on DM with a special emphasis on self-monitoring training. Our research results are consistent with those study findings.

Inadequate DK was reported in another study [18]; specifically, patients had poor knowledge related to diabetic diet (43.42%). In our study, apart from poor DK, patients also had poor knowledge related to diabetic diet, as only 33.1% correctly answered the relevant questions. questions regarding it. Another study from Nigeria [5] reported that more than 50% of the patients had poor DK, in comparison to our study, 71.43% of the diabetes patients had poor DK. Our study findings also showed that type 2 diabetics had poor DK regarding carbohydrates (33.33%), whereas, they had good knowledge regarding lipids and fats (50%) and proteins (50%), which contradicts the result of a study conducted by Sheard et al. [19].

Joshi and Joshi [20] recommended that brown bread or whole wheat bread, pasta, basmati rice, and potatoes should be the main part of meals for type 2 diabetics. In our research, only 28.8% of patients knew that dietary fiber is important in maintaining good glycemic control. The study conducted by Savoca and Miller [9] stated that patients’ food type and dietary behaviors might be influenced by good knowledge about diabetic diet recommendations. Our study results also showed that type 2 diabetics had good knowledge regarding food types (50%), and that this may have a positive influence on dietary practices.

The role of diet in controlling diabetes is considered imperative, however, diabetes patients are unaware how they should approach this issue. It is evident from the literature that nutrition intervention plays a very important role in providing diet-related self-management education to diabetes patients. This objective can be achieved through trained dietitians. However, to the best of our knowledge, dietitians are not available in any primary healthcare center (PHC) in Almajmaah City.

The American Diabetes Association (ADA) states that there is not a “one-size-fits-all” eating pattern for individuals with diabetes. Therefore, a patient empowerment approach should be used to counsel patients with poor DK. Counselling through an empowerment approach can help patients in managing their diabetes in a more effective way. Moreover, counselling should be in two parts, first by primary care physicians and secondly, by expert dietitians. The primary care physician should assess patients’ DK, identify = barriers, and empower patients to adopt such strategies that can improve their knowledge related to diabetic diet. After this, patients should be referred to dietitians where they should have individualized, patient-centered dietary education sessions. To achieve the maximum benefit out of these sessions, the dietitian should have knowledge of the cultural beliefs, thoughts, family, and communal networks of the patients.

Special emphasis should be given to the role of diet in diabetes awareness programs to carry out diet-related self-management in an efficient way. Strychar et al. [21] highlighted the importance of diet self-efficacy in the management of type 2 diabetes mellitus (T2DM). Out of 19 articles that met the inclusion criteria, only three studies focused on dietary self-efficacy. The authors emphasized the importance of addressing dietary self-efficacy using a patient empowerment approach, as this can have a positive effect on glycemic control. Furthermore, for educating and increasing awareness among people in the diabetes community, behavioral change communication material (BCCM) should be introduced, which should be pictorial, attractive, and appealing abrasive in the form of audio–visual presentations, side-by-side with print media.

The limitations of the study are that it has been conducted in only one region and results cannot be generalized to the other regions of KSA. For some questions, more than one answer was correct and, therefore, chances for randomly picking the right answer(s) differs between the questions. Additionally, the study was conducted among T2DM patients without co-morbidities. Apart from these limitations, there are a number of strong points; the data were collected using a systematic random sampling technique, which falls under the auspices of a random sampling technique. The sample size was statistically calculated with an optimum number included in the study. The questionnaire used underwent psychometric analysis; thus, we used a valid and reliable questionnaire. As this was the first study that assessed and evaluated the DK of type 2 diabetics, these results can act as a baseline for similar studies conducted in the KSA and neighboring Gulf Cooperation Council (GCC) countries.

## 5. Conclusions

Type 2 diabetics have an overall inadequate dietary knowledge. Poor dietary knowledge was also observed related to the consumption of carbohydrates and food choices. The role of diet in controlling diabetes is considered imperative; however, diabetes patients are unaware of how they should approach this issue. A patient empowerment approach can be used to counsel patients with poor DK. Primary care physicians and dietitians should carry out an individualized, patient-centered dietary education sessions. In addition, diet related behavioral change communication material can also be introduced to educate and dispense awareness in the diabetic community.

## Figures and Tables

**Table 1:** Risk factor profile of patients (n = 350).

**Duration of diabetes**	**n (%)**	**Family history**	**n (%)**
<5 years 5–10 years 11–15 years >15 years	123 (35.1) 194 (55.4) 15 (4.3) 18 (5.1)	Yes No	142 (40.6) 208 (59.4)
**Mode of diagnosis**	**n (%)**	**Smoking**	n (%)
Routine checkup At screening Symptomatic Emergency	242 (69.1) 44 (12.6) 38 (10.9) 26 (7.4)	Yes No Never-smokers	141 (40.3) 187 (53.4) 22 (6.3)
**Treating doctor**	**n (%)**	**Body Mass Index**	n (%)
General practitioner Physician Endocrinologist	262 (74.9) 74 (21.1) 14 (4.0)	Underweight Normal weight Overweight Obese	12 (3.4) 56 (16.0) 167 (47.7) 115 (32.9)

**Table 2:** Dietary knowledge of type 2 diabetics (n = 350).

Item	Stem	n (%) Correct Answers
1	The diabetics’ diet is?	116 (33.1)
2	Which of the following is highest in carbohydrates?	121 (34.6)
3	Which of the following is highest in fat?	150 (42.9)
4	Which of the following is a food which you can eat freely?	155 (44.3)
5	What is the effect of unsweetened fruit juice on blood glucose?	141 (40.3)
6	Which should be used to treat low blood sugar?	119 (34.0)
7	A well-balanced diet includes?	178 (50.9)
8	Which of these foods has amount of sugar in them?	148 (42.3)
9	Which of these foods has highest amount of fat in them?	127 (36.3)
10	HbA1c test has some relationship with your diet?	133 (38.0)
11	Which of the following contains highest amount of proteins?	145 (41.4)
12	Food that contains fats and oils gives us a lot of?	132 (37.7)
13	Eating too much sugary food may cause?	169 (48.3)
14	Which of the following foods contains most cholesterol?	125 (35.7)
15	Breads, cereals, rice, and pasta are high in?	151 (43.1)
16	Whole-grain foods like brown rice and whole wheat bread are better choices than white rice and white bread because whole-grains contains?	99 (28.3)
17	Which of the following foods is a complete source of protein?	175 (50.0)
18	Most of the excess sodium in our diet comes from?	100 (28.6)
19	Which of the following has the highest glycemic index?	170 (48.6)
20	Which of the following contains a vitamin that is needed for good vision?	153 (43.7)
21	Do you think that justified consumption of vitamin, mineral, carbohydrates, fat and protein have direct effect on outcomes of diabetes mellitus?	134 (38.3)

**Table 3:** Sub-groups of dietary knowledge.

No	Stem	n (%) Correct Answers
**Carbohydrates**		
1	Which of the following is highest in carbohydrates?	121 (34.6)
2	What is the effect of unsweetened fruit juice on blood glucose?	141 (40.3)
3	Which of these foods has amount of sugar in them?	148 (42.3)
4	Eating too much sugary food may cause?	169 (48.3)
5	Breads, cereals, rice, and pasta are high in?	151 (43.1)
6	Whole-grain foods like brown rice and whole wheat bread are better choices than white rice and white bread because whole-grains contains?	99 (28.3)
	**Lipids and Fats**	
1	Which of the following is highest in fat?	150 (42.9)
2	Which of these foods has highest amount of fat in them?	127 (36.3)
3	Food that contains fats and oils gives us a lot of?	132 (37.7)
4	Which of the following foods contains most cholesterol?	125 (35.7)
**Proteins**		
1	Which of the following contains highest amount of proteins?	145 (41.4)
2	Which of the following foods is a complete source of protein?	175 (50.0)
**Food Type**		
1	A well-balanced diet includes?	178 (50.9)
2	HbA1c test has some relationship with your diet?	133 (38.0)
3	Most of the excess sodium in our diet comes from?	100 (28.6)
4	Which of the following has the highest glycemic index?	170 (48.6)
5	Which of the following contains a vitamin that is needed for good vision?	153 (43.7)
6	Do you think that justified consumption of vitamin, mineral, carbohydrates, fat and protein have direct effect on outcomes of diabetes mellitus?	134 (38.3)
**Food Choices**		
1	The diabetics’ diet is?	116 (33.1)
2	Which of the following is a food which you can eat freely?	155 (44.3)
3	Which should be used to treat low blood sugar?	119 (34.0)

## Supplementary Materials

The following are available online at https://www.mdpi.com/1660-4601/17/3/858/s1.
