# Supplementary material for: Dietary Knowledge among Adults with Type 2 Diabetes—Kingdom of Saudi Arabia
Source: Int J Environ Res Public Health. 2020 Jan 30;17(3):858. doi: 10.3390/ijerph17030858 (PMC7037964; doi:10.3390/ijerph17030858)
Supplement: Supplementary file 1 [file ijerph-17-00858-s001.pdf]

Questionnaire

1. The diabetics' diet is:
   a. The way most people eat.
   b. A healthy diet for most people.
   c. Too high in carbohydrates for most people.
   d. Too high in protein for most people.
2. Which of the following is highest in carbohydrates?
   a. Baked chicken.
   b. Cheese.
   c. Baked potato.
   d. Peanut butter.
3. Which of the following is highest in fat?
   a. Low fat milk.
   b. Orange juice.
   c. Corns.
   d. Honey.
4. Which of the following is a food which you can eat freely?
   a. Any un-sweetened food.
   b. Any dietic food.
   c. Any food that says, "sugar free" on label.
   d. Any food that has less than 20 calories per serving.
5. What is the effect of un-sweetened fruit juice on blood glucose?
   a. Lowers it.
   b. Raises it.
   c. Has no effect.
6. Which should be used to treat low blood sugar:
   a. 3 hard candies.
   b. ½ cup orange juice.
   c. 1 cup diet soft drink.
   d. 1 cup skimmed milk.
7. A well-balanced diet includes:
   a. Green leafy vegetables.
   b. Fiber rich foods.
   c. Low sugar, oil and fat.
   d. High sugar, salt and oil.
8. Which of these foods has highest amount of sugar in them?
   a. Fruit juices.
   b. Potatoes.
   c. Artificial sweeteners.
   d. Diet soft drinks.
9. Which of these foods has highest amount of fat in them?
   a. Butter / Margarine.
   b. Brown bread.
   c. Chicken.
   d. Rice.
10. HbA1c test has some relationship with your diet?
   a. No relation.
   b. Strong relation.
   c. It's just a routine test.
11. Which of the following contains highest amount of proteins?
   a. Chicken and meat.
   b. Fruits and vegetables.
   c. Pulses and rice.
   d. Cereals.
12. Food that contains fats and oils gives us a lot of?

a. Vitamins.
b. Calories.
c. Minerals.
13. Eating too many sugary foods may cause?
a. Diabetes.
b. Hypertension.
c. Tooth decay.
d. Heart disease.
14. Which of the following foods contains most cholesterol?
a. Peanut butter.
b. Chicken.
c. Strawberry jam.
d. Chocolate spread.
15. Breads, cereals, rice, and pasta are high in?
a. Calcium.
b. Carbohydrates.
c. Fat.
d. Protein.
16. Whole-grain foods like brown rice and whole wheat bread are better choices than white rice and white bread because whole-grains contains:
a. More fiber.
b. More vitamin C.
c. Less minerals.
d. More carbohydrates.
17. Which of the following foods is a complete source of protein?
a. Bread.
b. Fish.
c. Dried beans.
d. Doughnut.
18. Most of the excess sodium in our diet comes from?
a. Salt we add at the table.
b. Sodium that is added during food processing and preparation.
c. Sodium that occurs naturally in foods.
19. Which of the following has the highest glycemic index?
a. Ice cream.
b. Cucumber.
c. Bread.
d. Dextrose.
20. Which of the following contains a vitamin that is needed for good vision?
a. Carrot.
b. Apple.
c. Mango.
d. Cucumber.
21. Do you think that justified consumption of vitamin, mineral, carbohydrates, fat and protein have direct effect on outcomes of diabetes mellitus?
a. Yes.
b. No.
c. I don’t know.
